# Efficacy and Safety of Topical Dapsone in Dermatology: A Scoping Review of Clinical Studies

**DOI:** 10.1111/jocd.70494

**Published:** 2025-10-06

**Authors:** Bahareh Abtahi‐Naeini, Hossein Sattari, Kimia Afshar, Fereshte Rastegarnasab, Mahsa Pourmahdi‐Boroujeni

**Affiliations:** ^1^ Pediatric Dermatology Division, Department of Pediatrics Imam Hossein Children's Hospital, Isfahan University of Medical Sciences Isfahan Iran; ^2^ Skin Diseases and Leishmaniasis Research Center Isfahan University of Medical Sciences Isfahan Iran; ^3^ Clinical Research Development Center, Najafabad Branch Islamic Azad University Najafabad Iran; ^4^ Student Research Committee Isfahan University of Medical Sciences Isfahan Iran; ^5^ Isfahan Eye Research Center Isfahan University of Medical Sciences Isfahan Iran

**Keywords:** dapsone, dermatology, skin, sulfones, topical

## Abstract

**Background:**

Dapsone (4,4′‐Diamino diphenyl sulfone) has been utilized in managing a wide variety of mucocutaneous conditions, usually as a systemic product. Topical dapsone is commercially available as 5% and 7.5% gel and is FDA‐approved for acne vulgaris. These topical formulations have shown less systemic absorption and fewer adverse events. Considering this, topical dapsone might benefit localized disease, immunosuppressed individuals, and those who avoid exposure to systemic therapies.

**Methods:**

Following PRISMA guidelines, a comprehensive literature search was conducted across PubMed, Web of Science, and Scopus databases for published papers up to April 2025. Then, eligible studies are included, and we summarize the findings of different clinical studies concerning the use, efficacy, and side effects of topical dapsone in various mucocutaneous conditions, excluding acne vulgaris.

**Results:**

Finally, 56 articles were selected based on the eligibility criteria. We summarized and categorized them into two general entities, including applications of the topical dapsone and side effects of the topical dapsone. The first entity has six subheadings consisting of acne vulgaris and acneiform eruptions, Rosacea, neutrophilic dermatosis, vesiculobullous diseases, vasculitis, and others.

**Conclusion:**

In conclusion, topical dapsone appears to have therapeutic advantages in several mucocutaneous conditions, especially in minimal, localized, and chronic eruptions.

## 1 Introduction

Dapsone is a sulfone compound widely recognized for its bacteriostatic properties, specifically inhibiting leprosy bacilli. In addition, dapsone is recognized for its anti‐inflammatory and antioxidant properties without immunosuppressive characteristics. Orally administered dapsone is FDA‐approved for treating leprosy, dermatitis herpetiformis, and acne vulgaris (AV). It is also prescribed for a wide range of skin diseases that involve neutrophils and eosinophils in their pathogenesis, including neutrophilic dermatoses, autoimmune bullous disorders, granulomatous inflammation, vasculitis, cutaneous lupus erythematosus, and prurigo pigmentosa [1, 2, 3].

Dapsone's antimicrobial properties stem from its ability to inhibit dihydrofolate reductase, thereby hindering dihydrofolic acid synthesis. Its anti‐inflammatory and antioxidant effects are achieved through scavenging oxygen radicals, impairing the chemotaxis of human neutrophils, diverting beta‐2 integrin (CD11b/CD18)‐mediated neutrophil adherence, and downregulating interleukin‐8 (IL‐8). Dapsone can limit the accumulation of neutrophils and eosinophils in inflamed tissues and inhibit myeloperoxidase. Furthermore, it exhibits dose‐dependent inhibition of TNF‐α and leukotriene product generation in leukocytes. Dapsone can decrease histamine release by reducing the impact of eosinophil peroxidase on mast cells [3, 4, 5].

However, dapsone is associated with various dose‐dependent and idiosyncratic side effects. Common adverse effects include nonspecific gastrointestinal symptoms, headache, nervousness, and abnormalities in liver function. Serious issues such as methemoglobinemia, hemolysis, fatal agranulocytosis, peripheral neuropathy, and blindness have also been reported. The anti‐inflammatory role of dapsone is generally dose‐dependent, with higher doses providing more intensive effects. However, the risk of dapsone‐dependent side effects increases if plasma concentration exceeds 5 mg/L. Dapsone can cross the placenta and be present in human milk, and the FDA categorizes it as a Category C drug in pregnancy [2, 3, 4].

Recent advancements have led to the introduction of a hydrogel product of dapsone. Dapsone gel, characterized by minimal systemic absorption, has emerged as a safe and effective alternative treatment, gaining widespread prescription [2]. Dapsone 5% and 7% gel have been FDA‐approved since 2005 and 2016, respectively, and are well recognized in AV management [1]. It is generally well tolerated, with mild local side effects. While isolated reports of methemoglobinemia following topical use exist, no severe reactions such as toxic epidermal necrolysis (TEN) or peripheral neuropathy have been reported [1, 6].

There are few reports supporting the use of topical dapsone in other mucocutaneous conditions. Its application may benefit patients with localized diseases, those seeking to avoid exposure to systemic therapies, or individuals who are already immunosuppressed [1, 2]. Nevertheless, topical dapsone use, regarding its properties, is quite underrated.

To the best of our knowledge, there is a notable absence of comprehensive studies regarding the off‐label clinical use of topical dapsone and its associated complications. This paper aims to summarize the findings related to the use, efficacy, and side effects of topical dapsone in various mucocutaneous conditions beyond its typical use in AV.

## 2 Methods

### 2.1 Design

The search methodology adhered to the Preferred Reporting Items for Systematic Reviews and Meta‐Analyses (PRISMA) protocol, ensuring a systematic search and review process to investigate the use, efficacy, and side effects of topical dapsone in diverse mucocutaneous conditions. Also, the protocol for this study was approved by the Sciences and Ethics Committee of Isfahan University of Medical Sciences (IR.ARI.MUI.REC.1402.211).

### 2.2 Eligibility Criteria

Clinical studies that reported using topical dapsone for mucocutaneous conditions in humans, including randomized controlled trials (RCTs), cohorts, case reports, and case series, were considered eligible for inclusion in this review. Studies that used dapsone in combination with other drugs were also considered eligible to be included regarding few studies on this subject. Articles that did not use topical formulations of dapsone, pharmacological (pharmacokinetics) studies, in vitro studies, conference papers, review articles, and unpublished studies were excluded. Studies investigating topical dapsone in AV were excluded from the results.

### 2.3 Information Sources and Search Strategy

The search strategy involved the selection of relevant MeSH terms, encompassing dapsone, 4,4′‐diaminodiphenyl sulfone, diaminodiphenylsulfone, and various topical formulations such as gel, ointment, cream, lotion, paste, emulsion, and drop. Following team discussions, definition, and formulation of search lines, a comprehensive search was conducted on April 25, 2025, across online databases, including PubMed, Scopus, and Web of Science. All articles containing the selected keywords in their title or abstract were included without any restriction on publication time.

### 2.4 Selection Process

The Zotero software was utilized to administrate and organize references during the writing and submission process of this review. Duplicate articles were removed from the final list. The articles were selected independently by two authors based on the title and abstract (K.A. and H.S.). In cases of disagreement between these two authors, a third author made the final decision (M.P.‐B.). Efforts were made to obtain the full text of all included articles, with requests sent to the corresponding authors as necessary. Additionally, the reference lists of the included papers underwent manual review to prevent overlooking any relevant publications not identified in the searches.

### 2.5 Data Collection

The data collection process encompassed several general characteristics of each study, including the first author's name, the publication year, and the study design. It also provided specific details about the study population, including the number of participants, their ages, and their sex. Additionally, information on the medical condition being treated, the treatment protocol, and a brief description of the main outcome was collected.

## 3 Results

**FIGURE 1:**
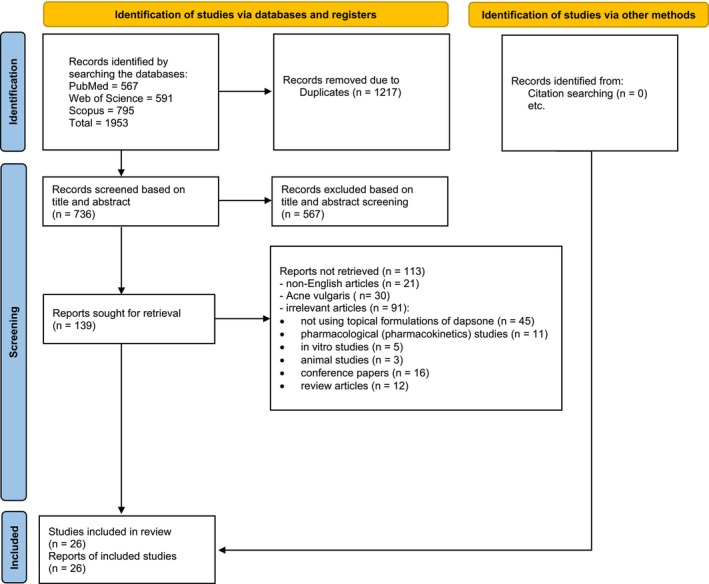
Flow‐chart of identifying related articles to the clinical use of topical dapsone.

Following the systematic search, a total of 1953 articles were identified. After eliminating duplicated records, 736 articles remained, and 567 were excluded based on irrelevant titles or abstracts. The full text of 169 articles underwent screening, resulting in the selection of 56 articles for inclusion. Figure 1 illustrates the PRISMA flow diagram, displaying the selection process (Figure 1).

A comprehensive summary of studies with applications of topical dapsone in mucocutaneous conditions rather than AV and cases with severe adverse effects, specifically methemoglobinemia, is shown in Table 1.

**TABLE 1:** Studies characteristics – topical dapsone use in various mucocutaneous conditions (except acne vulgaris).

Authors/Year	Disease/Study design/Study population	Participants characteristics (sex/age)	Medications	Dapsone prescription and time of consumption	Outcome
Barea‐Jiménez et al. [7] 2020	Toxic epidermal necrolysis/Case report/1	F/17	IVIG + S. C.S. + Palliative treatments + T. dapsone	5% three times daily	Clinically successful outcome
Handler et al. [8] 2010	Dermatitis herpetiform/Case report/1	M/14	S. dapsone + T. dapsone	5% twice daily NM	Clinically successful outcome within 4 weeks of treatment
Burbidge et al. [6] 2016	Dermatitis herpetiform/Case report/1	F/66	Gluten‐free diet + Topical dapsone	5% twice daily, for more than 1 year	The disease remained controlled with topical dapsone and a gluten‐free diet
Cinats et al. [9] 2019	Dermatitis herpetiform/Case report/1	F/44	Gluten‐free diet + Topical dapsone	5% twice daily NM	The disease remained controlled with topical dapsone and a gluten‐free diet. dapsone discontinued with no relapses
Handler et al. [10] 2011	Peristomal pyoderma gangrenosum/Case report/1	M/27	T. crushed dapsone	Crushed dapsone once daily, For 6 months	Treatment with topical application of crushed dapsone resulted in improvement of pyoderma gangrenosum and total lesion resolution after 6 months
Li et al. [11] 2018	Pyoderma gangrenosum Retrospective/Case series/21	15 F Participants/Mean = 60.3 (16.6)	T. dapsone + Other various medications	5% NM	Adjunct topical dapsone was associated with 85.7% partially and 9.5% completely improvement in pyoderma gangrenosum
Crouse et al. [12] 2018	Pyoderma gangrenosum/Case report/1	F/11 months	Steroids + T. tacrolimus + T. dapsone + T. clobetasol + S. infliximab Final treatment: S. tacrolimus + S. infliximab	5% once daily, for 6 days on dapsone	The disease was uncontrolled with topical dapsone and other topical treatment. Finally treated systemic tacrolimus and infliximab
Doolan et al. [13] 2019	Subcorneal pustular dermatosis/ Case report/1	F/82	S. dapsone + T. tacrolimus + T. betamethasone Dipropionate + T. dapsone	7.5% once daily NM	Systemic dapsone caused HB level decreased. Other therapy was unsuccessful. Topical dapsone in 3 weeks was associated with complete remission
Broussard et al. [14] 2012	Erosive pustular dermatosis/Case series/4	M/50	S. dapsone + T. dapsone	5% twice daily NM	The disease was completely treated in 17 weeks with topical and systemic dapsone with no recurrence
		F/90	T. dapsone	5% twice daily NM	The disease was completely treated with topical dapsone in 11 weeks with no recurrence
		F/51	T. dapsone	5% twice daily NM	The disease was completely treated in 10 weeks with topical dapsone with no recurrence
		M/83	T. dapsone	5% twice daily NM	The disease was completely treated with topical dapsone in 4 weeks with no recurrence
Melian‐Olivera et al. [15] 2022	Folliculitis decalvans/ Retrospective cohort/14	8 F Participants/ 32–62, Mean = 42	S. antibiotics needed in flares + T. dapsone	5% three times a week 7–54 months, Mean = 30 months	The total number of flare‐ups and months on oral antibiotics reduced significantly after admission of topical dapsone
Trüeb et al. [16] 2023	Folliculitis decalvans/ Commentary/1	—	T. dapsone	5% daily 6 weeks	The treatment resulted in worsening of FD with a pustular flare‐up. They advocated stricter criteria in trials to avoid clinical disappointments
Edek et al. [17] 2024	Neutrophilic dermatosis of the dorsal hands/Case Report/1	M/60	T. dapsone (Systemic immunosuppressive due to kidney transplantation)	5% twice daily NM	The disease was completely treated with topical dapsone in 4 weeks with no recurrence
Frieling et al. [18] 2013	Erythema elevatum diutinum/Case Report/1	M/81	S. dapsone + Topical dapsone	5% twice daily NM	Partially treated with topical dapsone and completely treated with adding systemic dapsone
Pate et al. [19] 2017	Leukocytoclastic vasculitis/Case report/1	F/50	Doxycycline + Amoxicillin + T. Triamcinolone + T. dapsone	5% three times a week NM	Other various treatment was unsuccessful but topical dapsone was associated with favorable response within 3 weeks
Aflatoonian et al. [20] 2016	Cutaneous Leishmaniasis/RCT/68	35 F Participants/AR = 7–80 dapsone: 31.6 (20.4) Cryotherapy: 29.6 (15.9)	Intralesional meglumine antimoniate + T. Niosomal dapsone vs. Intralesional meglumine antimoniate + Cryotherapy	NM, twice daily, for 16 weeks	Higher rate of completely treated in the dapsone group
Faghihi et al. [21] 2015	Papulopustular Rosacea/RCT/56	≥ 18	S. Doxycycline + T. dapsone VS. S. Doxycycline + T. Metronidazole	5% twice daily, for 12 weeks	Topical dapsone is a treatment option for papulopustular Rosacea, especially for those who suffer from pruritus and do not have significant dryness
Özkoca et al. [22] 2025	Papulopustular Rosacea/prospective observational study/32	32 F Participants/(18–70) Mean = 38.74 (13.08)	T. dapsone	7.5% once daily, for 8 weeks	The 7.5% topical dapsone formulation is effective and safe in treating papulopustular rosacea across all age groups
Ferguson et al. [23] 2019	Acne agminata/Case report/1	M/31	T. dapsone	5% twice daily, For 11 months	After 6 months of treatment, the patient experienced recurrence of the disease and after 11 months, the treatment was successfully stopped with no recurrence
Gökşin et al. [24] 2024	Erythematotelangiectatic rosacea/CT/35	Median = 38 (19–62)	T. dapsone	5% twice daily, For 12 weeks	Topical dapsone is an effective and well tolerated treatment option for erythematotelangiectatic rosacea
Sheu et al. [25] 2016	Pustular psoriasis/Case Series/1	F/69	T. dapsone, Topical tazarotene, Topical steroids, NBUVB	5% twice daily, For 18 months	The treatment was Continued on 5% topical dapsone with disease control (In this case series only one of the female cases was treated with topical dapsone)
Babalola et al. [26] 2014	Granuloma faciale/Case report/1	M/68	Various previous unsuccessful treatment Final treatment: T. dapsone	5% twice daily, For 18 months	A 50% clinical improvement was observed after 6 months and near‐complete resolution after 9 months. At an 18‐month follow‐up, the plaque had nearly regressed
Kassardjian et al. [27] 2015	Granuloma annulare/Case report/1	M/41	T. dapsone	5% twice daily, For 3 weeks	The patient demonstrated significant clinical improvement after 3 weeks of applying topical dapsone
Watton et al. [28] 2015	Methemoglobinemia/Case Report/1	F/19	T. dapsone	5% twice daily, For 7 days	The patient developed methemoglobinemia after applying topical dapsone for 7 days
Graff et al. [29] 2016	Methemoglobinemia/Case Report/1	F/19 months	T. dapsone	5%, Only one time applying	The patient developed methemoglobinemia after applying a single dose of topical dapsone the night before admission
Yale et al. [30] 2020	Methemoglobinemia/Case report/1	F/15	T. dapsone	7.5% once daily, For 2 weeks	The patient developed methemoglobinemia after applying topical dapsone to her face, chest, and back daily for 2 weeks despite the prescription being written for facial use only
Cantrel et al. [31] 2022	Methemoglobinemia/Case report/1	F/15	T. dapsone	NM, For 2 days	The patient developed methemoglobinemia after applying topical dapsone for 2 days

Abbreviations: F, female; M, male; NM, not mentioned; RCT, randomized clinical trial; S. at the initiation of the drug name, Systemic formulation of the drug; T. at the initiation of the drug name, Topical formulation of the drug; VS, versus.

## 4 Discussion

**FIGURE 2:**
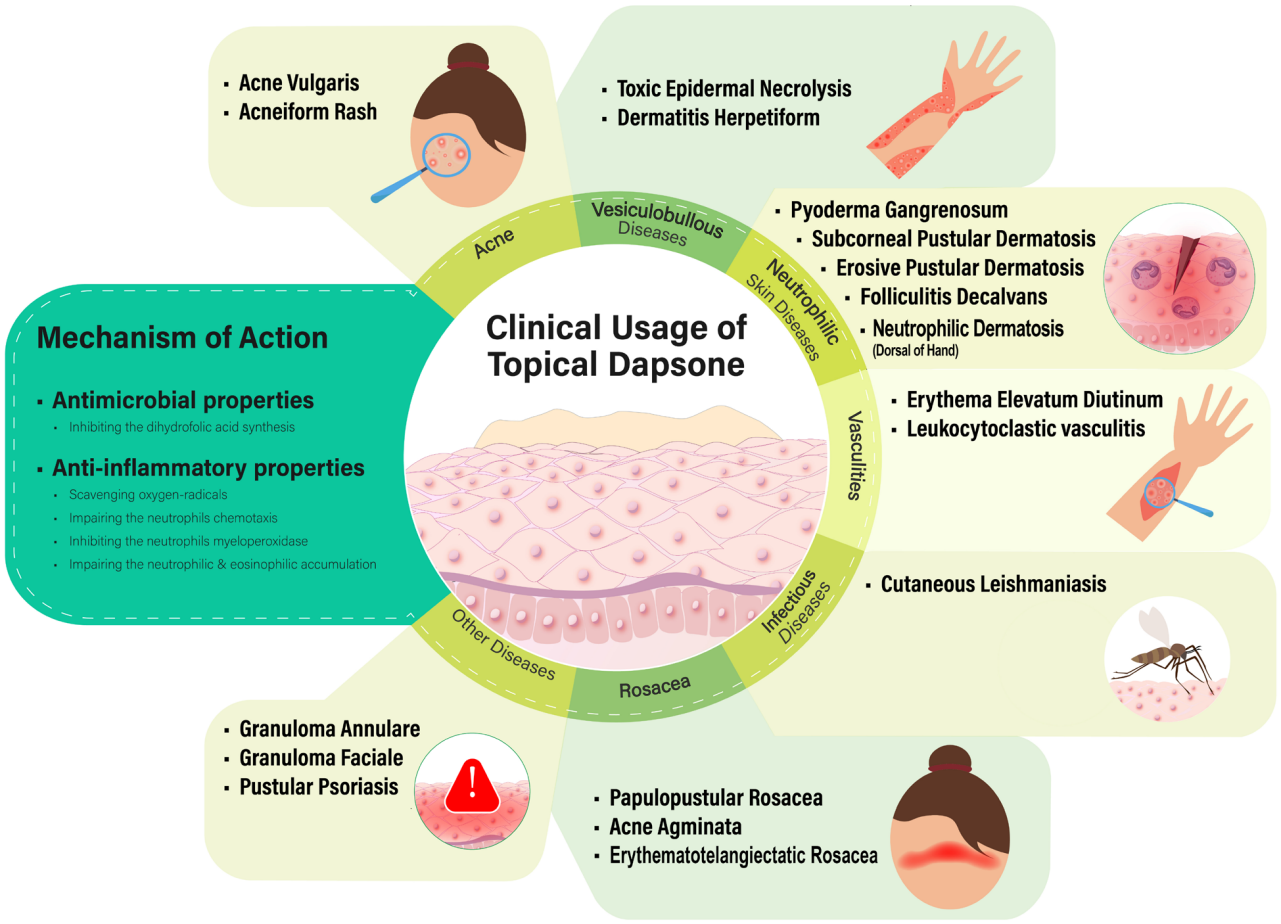
A summary of the mechanism of action and clinical usages of topical dapsone.

The findings are categorized into two broad entities: (1) applications of topical dapsone and (2) side effects of topical dapsone. The first entity comprises six subheadings, as summarized in Figure 2, encompassing (1.1) acne vulgaris, (1.2) Rosacea, (1.3) neutrophilic dermatosis, (1.4) vesiculobullous Diseases, (1.5) vasculitis, and (1.6) others (Figure 2).

### 4.1 Use of Topical Dapsone in Different Diseases

#### 4.1.1 Topical Dapsone in Acne Vulgaris

Dapsone gel, either 5% or 7.5%, was FDA‐approved in 2005 and 2016, respectively, for clinical indications, specifically AV. The efficacy and safety of the drug have been investigated in several studies. Most of the included studies (30 separate studies) have demonstrated dapsone gel as an effective and safe treatment option for AV. Several reviews have already provided comprehensive overviews on this topic. Therefore, we decided to omit an in‐depth discussion of this aspect from the current study. A summary of the search results is presented in Table S1.

#### 4.1.2 Topical Dapsone in Vesiculobullous Diseases

##### 4.1.2.1 Toxic Epidermal Necrolysis/Steven Johnson Syndrome

A 17‐year‐old female patient developed TEN after taking amoxicillin; in addition to the standard treatment of Intravenous Immunoglobulin (IVIG) and systemic corticosteroids, she was prescribed dapsone gel 5% three times a day for her intraoral lesions, which resulted in a successful outcome. The authors suggested that using dapsone gel with palliative treatments could be a promising alternative for addressing oral lesions in TEN/Stevens‐Johnson syndrome [7].

##### 4.1.2.2 Dermatitis Herpetiform

Handler et al. reported the successful treatment of a 14‐year‐old boy with DH using topical dapsone 5%. Initial treatment involved oral dapsone at 25 mg, subsequently increased to 50 mg daily due to gluten‐free diet intolerance. Furthermore, dapsone gel 5% was prescribed and applied unilaterally to ascertain the most effective treatment. A 4‐week follow‐up revealed superior clinical results on the topical dapsone‐treated side [8].

Burbidge et al. presented the case of a 66‐year‐old individual with DH who was prescribed topical gel 5% and adhered to a gluten‐free diet. Within 2 weeks of dapsone use and 3 days of complying with the gluten‐free diet, lesion regression occurred. Notably, her condition remained controlled solely with topical dapsone for over a year [6].

In an additional case study, a 44‐year‐old woman with a history of Hashimoto thyroiditis and DH was treated with topical 5% gel and a gluten‐free diet. Cutaneous lesions cleared within 3 days, and maintaining a gluten‐free diet prevented further clinical episodes. Eventually, dapsone was discontinued without complications [9].

#### 4.1.3 Topical Dapsone in Neutrophilic Skin Diseases

##### 4.1.3.1 Pyoderma Gangrenosum

A survey was conducted from 2000 to 2015 on 21 patients who were prescribed topical dapsone in combination with other drugs for their PG; 85.7% of the patients partially improved, and 9.5% completely improved. Based on these findings, the authors suggest topical dapsone as an adjunct treatment in PG. This low‐risk, steroid‐sparing treatment may benefit patients with PG, especially those who avoided exposure to systemic therapies or who are already immunosuppressed [11].

Crouse et al. report a case of infantile PG that remained uncontrolled despite treatment with various topical and systemic medications, including steroids, topical tacrolimus, clobetasol ointment, and infliximab infusions. Topical dapsone also was ineffective in this case and failed to provide adequate disease control. However, a combination treatment of systemic tacrolimus and infliximab infusions was effective in achieving remission for 9 months without adverse effects. This case highlights the importance of considering alternative treatments when topical therapies like dapsone gel are insufficient [12].

Additionally, Handler et al. reported a 27‐year‐old man with a 6‐year history of peristomal pyoderma gangrenosum and Crohn's disease. He previously received intralesional or systemic corticosteroids and systemic dapsone but discontinued them due to elevated transaminase levels. Therefore, he was prescribed daily self‐crushed 25 mg dapsone tablets with dressing changes. Within 1 month, the lesion started to regress, and in 6 months, it resolved with no recurrences in follow‐up [10].

##### 4.1.3.2 Subcorneal Pustular Dermatosis

In 2019, Doolan et al. reported a case study of an 82‐year‐old female patient presenting with localized Subcorneal Pustular Dermatosis (SPD) in the bilateral axillary region without any systemic involvement or coexistent conditions. Initially, the patient received treatment with oral dapsone at a dosage ranging from 25 to 75 mg daily, alongside topical tacrolimus. However, due to diminishing hemoglobin levels and bone marrow suppression, oral dapsone was discontinued. Subsequently, the patient utilized topical tacrolimus and betamethasone dipropionate ointment, resulting in a disease flare‐up. Ultimately, the prescription of topical dapsone 7.5% gel led to complete lesion remission within 3 weeks [13].

##### 4.1.3.3 Erosive Pustular Dermatosis

In a case series focusing on mild to moderate Erosive pustular dermatosis of the scalp (EPDS), four distinct cases resistant to multiple treatments, including tacrolimus, steroids, and antibiotics, were outlined. For 2 months, oral dapsone was administered to one of them, but the improvement was undesirable. However, upon the initiation of topical dapsone 5% gel twice daily, all four cases exhibited significant improvement in lesions without any signs of recurrence. The findings suggest that topical dapsone holds promise as a potential therapeutic option for managing scalp erosive pustular dermatosis [14].

##### 4.1.3.4 Folliculitis Decalvans

Given the neutrophilic component in folliculitis decalvans (FD), dapsone might be effective treatment options. A retrospective cohort study was conducted by Melián‐Olivera et al. to evaluate the efficacy of topical dapsone in 14 patients with FD. These patients applied topical dapsone gel 5% three times a week and were followed for 12 months. The clinical response was measured by the number of flare‐up episodes and the total number of months on oral antibiotics before and after the administration of topical dapsone. The results demonstrated a noteworthy decrease in both parameters following the use of dapsone (before = 0.13 flare‐ups per month; after = 0.03) [15].

However, Trüeb et al. challenged these findings in a commentary, reporting a case where 5% dapsone gel applied daily for 6 weeks resulted in worsening of FD with a pustular flare‐up. The authors questioned whether the positive outcomes in the Melián‐Olivera et al. study might reflect the natural progression of FD, potentially evolving to a less active phase over the extended mean treatment period (30 months), rather than a direct effect of dapsone. They advocated stricter criteria in dermatological treatment trials to avoid clinical disappointments [16].

##### 4.1.3.5 Neutrophilic Dermatosis of the Dorsal Hands

In another report, a 60‐year‐old male patient with neutrophilic dermatosis of the dorsal hands was treated with a 5% gel of dapsone twice daily to avoid the side effects of topical and systemic steroids. The reason for choosing this product was that the patient was already under an immunosuppressive regimen due to diabetes mellitus and kidney transplantation. Topical dapsone had a complete clinical response and no recurrence in a 1‐year follow‐up [17].

#### 4.1.4 Topical Dapsone in Vasculitis

##### 4.1.4.1 Cutaneous Vasculitis

While cutaneous vasculitis often resolves spontaneously, in rare cases, it can manifest as chronic or severe, necessitating systemic intervention. In a case reported by Frieling and colleagues, an 81‐year‐old man with a clinical diagnosis of erythema elevatum diutinum during the diagnostic confirmation interval was initially prescribed dapsone 5% gel. Partial improvement of lesions was noted during this period. Subsequently, upon confirmation of the diagnosis, oral dapsone was added, resulting in the complete resolution of lesions [18].

Pate et al., reported a 60‐year‐old woman with localized Leukocytoclastic vasculitis (LCV) on her lower extremities with no response to treatment to doxycycline and amoxicillin. She was also intolerant to systemic corticosteroids, so topical triamcinolone cream 0.1% was prescribed twice daily, but the disease progressed. After initiation of topical dapsone gel 5% twice daily, she experienced resolution of her previously refractory condition within 3 weeks [19].

#### 4.1.5 Topical Dapsone in Infectious Disease

##### 4.1.5.1 Cutaneous Leishmaniasis

Aflatoonian et al. conducted an RCT to assess the impact of niosomal dapsone gel in the management of cutaneous leishmaniasis (CL). Sixty‐eight participants previously diagnosed with leishmaniasis were randomized into two groups: one receiving biweekly cryotherapy and weekly intralesional meglumine antimoniate, and the other receiving topical dapsone twice daily in addition to weekly intralesional meglumine antimoniate. The dapsone‐treated group exhibited a complete response rate of 86.8%, while the comparison group demonstrated a rate of 82.9%. In 1 year of follow‐up, no recurrence occurred in the dapsone group. They suggested that niosomal dapsone gel can be an alternate and complementary treatment for treating CL [20].

#### 4.1.6 Topical Dapsone in Rosacea

##### 4.1.6.1 Papulopustular Rosacea

In 2015, Faghihi et al. conducted an RCT with 56 patients to evaluate the efficacy of dapsone compared to metronidazole gel in patients with papulopustular rosacea. Their investigation revealed that the improvement differences within groups were insignificant. Evaluation of adverse events among groups showed a lower rate of burning sensation in the dapsone group with a higher rate of scaling [21].

In a more recent study, Özkoca and Caf conducted a prospective study to assess the safety and efficacy of 7.5% dapsone gel in 32 female patients with papulopustular rosacea. The gel was applied once daily for 8 weeks. Significant reductions were observed in both lesion counts (from 22.1 to 3.87) and IGA scores (from 3.06 to 0.74) by week 8. No adverse effects were reported, and treatment efficacy was independent of age. The findings suggest that 7.5% topical dapsone is an effective and well‐tolerated monotherapy for papulopustular rosacea [22].

##### 4.1.6.2 Acne Agminata (Variant of Granulomatous Rosacea)

Ferguson et al. presented a 31‐year‐old man with asymptomatic facial papules that were clinicopathologically consistent with acne agminata. Some treatments include prednisolone, lymecycline, and isotretinoin, which are ineffective in controlling the disease. They initiated topical dapsone 5% twice daily. Six months later, dapsone was stopped intermittently, which led to a rapid recurrence and development of new lesions. Therefore, treatment continued for 11 months with complete clinical response and no recurrences. As a result, Authors suggest that topical dapsone can be prescribed as an effective, inexpensive, and tolerable maintenance option for AA [23].

##### 4.1.6.3 Erythematotelangiectatic Rosacea

In a study by Gökşin, topical dapsone was evaluated in erythematotelangiectatic rosacea. They enrolled 35 patients and prescribed 5% dapsone gel for 12 weeks twice daily. The results showed statistically significant improvement in investigator global assessment and visual analog scale. They concluded that dapsone was effective and tolerated in ETR treatment, but larger studies must be done [24].

#### 4.1.7 Topical Dapsone in Other Diseases

##### 4.1.7.1 Pustular Psoriasis

Sheu et al. reported on five cases of pustular psoriasis treated with dapsone, with one patient specifically utilizing dapsone gel 5%. Based on the favorable clinical responses observed in their patients, the authors suggested that dapsone, whether administered orally or topically, can be considered a viable treatment option for individuals with pustular psoriasis [25].

##### 4.1.7.2 Granuloma Faciale

In 2014, Babalola et al. documented the case of a 68‐year‐old man diagnosed with granuloma faciale (GF), who had undergone multiple unsuccessful treatments including intralesional triamcinolone, topical mometasone, and oral doxycycline. Following 6 months of utilizing dapsone gel 5% twice daily, a notable 50% clinical improvement was achieved. Considering the minimal side effects and high compliance associated with topical dapsone treatment, it should be considered as a noteworthy therapeutic option for GF [26].

##### 4.1.7.3 Granuloma Annulare

In a case reported by Kassardjian et al., a 41‐year‐old man with granuloma annulare (GA) experienced significant clinical improvement upon initiating treatment with dapsone gel 5%. The authors propose despite the various systemic and local therapeutic options available for GA, topical dapsone can be beneficial in the management of GA with a lower risk of infection and scarring [27].

### 4.2 Topical Dapsone Adverse Drug Reactions

The utilization of topical dapsone may be associated with mild adverse effects, including dryness, oiliness, erythema, peeling, sensitivity to touch, stinging, flaking, sun sensitivity, acne breakouts, tingling, tightness, nasopharyngitis, and headache. However, these side effects are generally insignificant and mild in the dapsone‐treated and vehicle‐treated groups. Studies have suggested that the emollient base of the vehicle gel itself can possess therapeutic value while potentially causing mild irritation [2, 32, 33].

One of the most noteworthy adverse effects of dapsone pertains to hematologic reactions, which impose limitations on the use of oral dapsone. However, topical formulations result in systemic exposure that is 100‐fold lower than that associated with oral dapsone treatment and exhibit minimal systemic absorption. Notably, among studies, no significant alterations in hemoglobin or other laboratory values were observed with topical dapsone, even among patients with G6PD deficiency [2, 14, 33]. Notably, topical dapsone is considered safe for patients with G6PD deficiency or sulfonamide allergies [1, 6].

Severe adverse events such as methemoglobinemia have been reported in limited cases, which are mentioned below:

#### 4.2.1 Methemoglobinemia

A 19‐year‐old female with a history of 1 week of applying a pea‐sized amount of dapsone 5% gel to her face twice daily presented to the emergency room with headache, dyspnea, and bluish discoloration of nails and lips, which was consistent with the diagnosis of methemoglobinemia. She was treated with a 100 mg single dose of methylene blue and discharged after 2 days [28].

In another report, Graff et al. introduced a 19‐month‐old girl admitted to the hospital with a blue‐like discoloration of skin, nails, and lips after applying an unknown amount of dapsone gel 5% to both her arms. The gel was prescribed for her brother due to AV. She was diagnosed with methemoglobinemia and was prescribed intravenous methylene blue. Her symptoms entirely resolved, and she was discharged after 2 days. It should be considered methemoglobinemia as a complication of topical dapsone, especially in young children [29].

A 15‐year‐old woman with a history of migraines was admitted to the hospital with anxiety, confusion, headache, dyspnea, and cyanosis. She applied dapsone gel 7.5% to her face, back, and chest once daily for 2 weeks, while she was prescribed to use only on her face. Workups revealed that the dapsone serum level was approximately twice the upper limit of average concentration for a patient who consumes a 200 mg dose of oral dapsone daily. After the second dose of methylene blue, cyanotic symptoms started to regress [30].

Cantrel et al. reported the case of a 15‐year‐old woman who developed methemoglobinemia after using topical dapsone for AV. Upon admission, she presented with symptoms of lightheadedness, nausea, palpitations, and progressively worsening dyspnea. Arterial blood gas with co‐oximetry revealed a methemoglobin level of 37.1%. Consequently, treatment with methylene blue at an initial dose of 1 mg/kg, ascorbic acid, and cimetidine was initiated. During her hospitalization, the methemoglobin level fluctuated significantly, stabilizing at less than 10% by the fourth day. She was discharged but returned to the emergency department on the same day, where a methemoglobin level of 16.9% was observed. Subsequent treatment with ascorbic acid resulted in a gradual decrease in her methemoglobin level to 0.6%. During the second admission, she developed normocytic anemia, leading to the initiation of prednisone for suspected autoimmune hemolytic anemia. On the 17th day after the initial admission, her clinical symptoms resolved, and her hemoglobin level stabilized. Despite an abnormality in the rheumatic panel, she was discharged and referred to a rheumatologist and hematologist [31].

This study has several limitations that need to be acknowledged. Most publications predominantly consisted of case reports. The limited number of studies on dapsone use and the wide variation in their methodologies resulted in significant heterogeneity among the included studies.

While the findings may suggest clinical efficacy, they also impose constraints on generalizability. Consequently, it is crucial to interpret the results, bearing in mind that further investigation, mainly through more robust methodological studies, is necessary for all clinical uses of dapsone rather than acne.

We conducted a literature review in the search stage and included research based on defined criteria. However, due to the restricted number of studies, a decision was made to include all relevant studies without conducting a quality assessment.

Furthermore, our examination was confined to English‐language publications, excluding articles in other languages, preprints, and conference papers. This might have caused an overrepresentation of favorable outcomes. Additionally, the sources of funding for the included studies were not disclosed in this current study, which, as a potential bias, can influence study outcomes. These limitations underscore the need for caution in drawing broad conclusions and highlight the necessity for more comprehensive and methodologically rigorous studies in the future.

## 5 Conclusion

Dapsone is a valuable treatment for mucocutaneous conditions, but topical formulations have been used primarily for AV. However, considering the topical dapsone's properties and advantages, it may be a practical choice in various conditions. This scoping review provided an overview of all clinical studies that used topical dapsone as their therapeutic process. We categorized them into six entities: acne vulgaris, rosacea, neutrophilic dermatosis, vesiculobullous diseases, vasculitis, and others. Despite few reports of possible methemoglobinemia, most reports were associated with promising outcomes, efficacy, and safety. We suggest larger‐scale and more robust methodological studies to investigate the efficacy and safety of topical dapsone in mucocutaneous conditions.

## Author Contributions

Bahareh Abtahi‐Naeini provided the concept of the study. Bahareh Abtahi‐Naeini, Mahsa Pourmahdi‐Boroujeni, and, Hossein Sattari, contributed to designing the study. Hossein Sattari assisted in data gathering. Mahsa Pourmahdi‐Boroujeni contributed to data analyzing. Fereshteh Rastegarnasab, and Kimia Afshar assisted in interpretation of the study's findings. Bahareh Abtahi‐Naeini, Mahsa Pourmahdi‐Boroujeni, and, Hossein Sattari, assisted in the preparation of the first draft of the manuscript and other authors have revised the manuscript critically for important intellectual content. All authors have read the final version and approved the content of the manuscript to be published and confirmed the accuracy or integrity of any parts of the work.

## Disclosure

Declaration of independency to government: We declare that none of the authors are employed by a government agency that has a primary function other than research and/or education. None of the authors have an official representative on behalf of the government.

## Conflicts of Interest

The authors declare no conflicts of interest.

## Supporting information


**Table S1:** Studies characteristics on the topic of topical dapsone in acne vulgaris and acneiform rashes.

## Data Availability

The data that support the findings of this study are available from the corresponding author upon reasonable request.

## References

[1] X. S. Wang , Z. Z. Wang , L. L. Sun , H. Liu , and F. R. Zhang , “Efficacy and Safety of Dapsone Gel for Acne: A Systematic Review and Meta‐Analysis,” Annals of Palliative Medicine 11, no. 2 (2022): 611–620. 35249339 10.21037/apm-21-3935

[2] E. Molinelli , M. Paolinelli , A. Campanati , V. Brisigotti , and A. Offidani , “Metabolic, Pharmacokinetic, and Toxicological Issues Surrounding Dapsone,” Expert Opinion on Drug Metabolism & Toxicology 15, no. 5 (2019): 367–379. 30943794 10.1080/17425255.2019.1600670

[3] G. Wozel and C. Blasum , “Dapsone in Dermatology and Beyond,” Archives of Dermatological Research 306, no. 2 (2014): 103–124. 24310318 10.1007/s00403-013-1409-7 PMC3927068

[4] N. Ghaoui , E. Hanna , O. Abbas , A. G. Kibbi , and M. Kurban , “Update on the Use of Dapsone in Dermatology,” International Journal of Dermatology 59, no. 7 (2020): 787–795. 31909480 10.1111/ijd.14761

[5] S. A. Temiz and M. Daye , “Dapsone for the Treatment of Acne Vulgaris: Do the Risks Outweigh the Benefits?,” Cutaneous and Ocular Toxicology 41, no. 1 (2022): 60–66. 34969324 10.1080/15569527.2021.2024565

[6] T. Burbidge and R. M. Haber , “Topical Dapsone 5% Gel as an Effective Therapy in Dermatitis Herpetiformis,” Journal of Cutaneous Medicine and Surgery 20, no. 6 (2016): 600–601. 27207357 10.1177/1203475416651053

[7] N. Barea‐Jiménez , J. Calero , D. Molina‐Negrón , L. M. López Del‐Valle , N. Barea‐Jimenez , and J. Calero , “Treatment for Oral Lesions in Pediatric Patients With Stevens‐Johnson's Syndrome: A Case Report and Literature Review,” International Journal of Paediatric Dentistry 30, no. 4 (2020): 489–496. 31923328 10.1111/ipd.12615

[8] M. Z. Handler , A. H. Chacon , M. I. Shiman , and L. A. Schachner , “Letter to the Editor: Application of Dapsone 5% Gel in a Patient With Dermatitis Herpetiformis,” Journal of Dermatology Case Reports 6, no. 4 (2012): 132–133. 10.3315/jdcr.2012.1124 PMC3543863 23329996

[9] A. K. Cinats , L. M. Parsons , and R. M. Haber , “Facial Involvement in Dermatitis Herpetiformis: A Case Report and Review of the Literature,” Journal of Cutaneous Medicine and Surgery 23, no. 1 (2019): 35–37. 30103636 10.1177/1203475418795818

[10] M. Z. Handler , H. Hamilton , and D. Aires , “Treatment of Peristomal Pyoderma Gangrenosum With Topical Crushed Dapsone,” Journal of Drugs in Dermatology 10, no. 9 (2011): 1059–1061. 22052278

[11] D. G. Li , R. S. Din , W. G. Tsiaras , and A. Mostaghimi , “Evaluating the Efficacy of Topical Dapsone Treatment for Pyoderma Gangrenosum: A Retrospective Case Series,” Journal of Cutaneous Medicine and Surgery 22, no. 6 (2018): 650–651. 30322308 10.1177/1203475418782140

[12] L. Crouse , D. McShane , D. S. Morrell , and E. Y. Wu , “Pyoderma Gangrenosum in an Infant: A Case Report and Review of the Literature,” Pediatric Dermatology 35, no. 5 (2018): E257–E261. 29656404 10.1111/pde.13471

[13] B. J. Doolan , W. C. Cranwell , J. Nicolopoulos , and C. Dolianitis , “Topical Dapsone Gel for Treatment of Axillary Subcorneal Pustular Dermatosis,” Journal of Dermatology 46, no. 11 (2019): e437–e438. 31232481 10.1111/1346-8138.14953

[14] K. C. Broussard , T. G. Berger , M. Rosenblum , and J. E. Murase , “Erosive Pustular Dermatosis of the Scalp: A Review With a Focus on Dapsone Therapy,” Journal of the American Academy of Dermatology 66, no. 4 (2012): 680–686. 22074698 10.1016/j.jaad.2011.10.011

[15] A. Melián‐Olivera , P. Burgos‐Blasco , G. Selda‐Enríquez , et al., “Topical Dapsone for Folliculitis Decalvans: A Retrospective Cohort Study,” Journal of the American Academy of Dermatology 87, no. 1 (2022): 150–151. 34246696 10.1016/j.jaad.2021.07.004

[16] R. M. Trüeb , N. N. C. Luu , and H. D. Rezende , “Comment on Topical Dapsone for Folliculitis Decalvans,” International Journal of Trichology 15, no. 3 (2023): 88–90. 38179006 10.4103/ijt.ijt_39_22 PMC10763727

[17] Y. C. Edek , F. Tamer , E. Adisen , and Ö. Erdem , “A Rapid and Complete Clinical Response to Topical Dapsone Treatment in a Patient With Neutrophilic Dermatosis of the Dorsal Hands: A Rare Case Report,” Dermatologica Sinica 42, no. 4 (2024): 301–302.

[18] G. W. Frieling , N. L. Williams , S. J. M. Lim , and S. I. Rosenthal , “Novel Use of Topical Dapsone 5% Gel for Erythema Elevatum Diutinum: Safer and Effective,” Journal of Drugs in Dermatology 12, no. 4 (2013): 481–484. 23652900

[19] D. A. Pate , L. S. Johnson , and M. B. Tarbox , “Leukocytoclastic Vasculitis Resolution With Topical Dapsone,” Cutis 99, no. 6 (2017): 426–428. 28686752

[20] M. Aflatoonian , A. Fekri , Z. Rahnam , et al., “The Efficacy of Combined Topical Niosomal Dapsone Gel and Intralesional Injection of Meglumine Antimoniate in Comparison With Intralesional Meglumine Antimoniate and Cryotherapy in the Treatment of Cutaneous Leishmaniasis,” Journal of Pakistan Association of Dermatologists 26, no. 4 (2016): 353–360.

[21] G. Faghihi , P. Khosravani , M. A. Nilforoushzadeh , et al., “Dapsone Gel in the Treatment of Papulopustular Rosacea: A Double‐Blind Randomized Clinical Trial,” Journal of Drugs in Dermatology 14, no. 6 (2015): 602–606. 26091386

[22] D. Özkoca and N. Caf , “The Treatment Efficacy of 7.5% Dapsone Gel in Papulopustular Rosacea: A Prospective Study,” Cutaneous and Ocular Toxicology 43, no. 4 (2024): 405–409. 39529605 10.1080/15569527.2024.2424932

[23] L. Ferguson and L. Fearfield , “Topical Dapsone Gel Is a New Treatment Option for Acne Agminata,” Clinical and Experimental Dermatology 44, no. 4 (2019): 453–455. 30246349 10.1111/ced.13742

[24] Ş. Gökşin , I. G. İmren , and N. Kaçar , “Efficacy of Topical Dapsone 5% Gel for the Treatment of Erythematotelangiectatic Rosacea: New Treatment Option With Old Drug,” Dermatology Practical & Conceptual 14, no. 1 (2024): 1–7. 10.5826/dpc.1401a34 PMC10868780 38364435

[25] J. S. Sheu , S. J. Divito , M. Enamandram , and J. F. Merola , “Dapsone Therapy for Pustular Psoriasis: Case Series and Review of the Literature,” Dermatology 232, no. 1 (2016): 97–101. 26465742 10.1159/000431171

[26] O. Babalola , J. Zhang , A. Kristjansson , D. Whitaker‐Worth , and M. McCusker , “Granuloma Faciale Treated With Topical Dapsone: A Case Report,” Dermatology Online Journal 20, no. 8 (2014): 13030. 25148282

[27] M. Kassardjian , M. Patel , P. Shitabata , and D. Horowitz , “Management of Periocular Granuloma Annulare Using Topical Dapsone,” Journal of Clinical and Aesthetic Dermatology 8, no. 7 (2015): 48–51. PMC4509586 26203321

[28] C. Watton , K. Smith , E. Carter , G. S. Swartzentruber , J. H. Yanta , and A. F. Pizon , “Methemoglobinemia as a Complication of Topical Dapsone,” New England Journal of Medicine 372, no. 5 (2015): 492. 10.1056/NEJMc1408272 25629755

[29] D. M. Graff , G. M. Bosse , and J. Sullivan , “Case Report of Methemoglobinemia in a Toddler Secondary to Topical Dapsone Exposure,” Pediatrics 138, no. 2 (2016): e20153186. 27401099 10.1542/peds.2015-3186

[30] S. Yale , N. Stefanko , P. McCarthy , V. McFadden , and J. McCarthy , “Severe Methemoglobinemia due to Topical Dapsone Misuse in a Teenage Girl,” Pediatric Dermatology 37, no. 2 (2020): 377–378. 31876314 10.1111/pde.14080

[31] C. Cantrell , V. Costers , C. C. Wilson , C. J. Dudek , and J. K. Arnold , “Refractory Methemoglobinemia Secondary to Topical Dapsone With Subsequent Autoimmune Hemolytic Anemia,” Cureus Journal of Medical Science 14, no. 9 (2022): 5. 10.7759/cureus.28811 PMC9534637 36225525

[32] J. Q. Del Rosso , L. Kircik , and C. J. Gallagher , “Comparative Efficacy and Tolerability of Dapsone 5% Gel in Adult Versus Adolescent Females With Acne Vulgaris,” Journal of Clinical and Aesthetic Dermatology 8, no. 1 (2015): 31–37. PMC4295856 25610522

[33] Z. D. Draelos , E. Carter , J. M. Maloney , et al., “Two Randomized Studies Demonstrate the Efficacy and Safety of Dapsone Gel, 5% for the Treatment of Acne Vulgaris,” Journal of the American Academy of Dermatology 56, no. 3 (2007): 439.e1‐10. 10.1016/j.jaad.2006.10.005 17208334

