# Supplementary material for: Efficacy and Safety of Topical Dapsone in Dermatology: A Scoping Review of Clinical Studies
Source: J Cosmet Dermatol. 2025 Oct 6;24(10):e70494. doi: 10.1111/jocd.70494 (PMC12498492; doi:10.1111/jocd.70494)
Supplement: Supplementary file 1 — Table S1: Studies characteristics on the topic of topical dapsone in acne vulgaris and acneiform rashes. [file JOCD-24-e70494-s001.docx]

*Table 1) Studies Characteristics – Acne Vulgaris and Acneiform Rashes*

| **Authors/**  **Year** | **Disease/Study Design/ Study Population** | **Participants characteristics** | **Medications** | **Dapsone prescription & Time of Consumption** | **Outcome** |
| --- | --- | --- | --- | --- | --- |
| Draelos, et al. ^(1)^  2007 | Acne Vulgaris  RCT  3010 | Dapsone: 12-81, Mean = 19.3 (7.5)  Vehicle: 11-59, Mean = 19.6 (7.6) | T. Dapsone VS. T. Vehicle | 5% twice daily, for 12 weeks | Dapsone gel was an effective, safe, and well-tolerated treatment for AV with a rapid onset of response |
| Lucky, et al. ^(2)^  2007 | Acne Vulgaris  RCT  486 | ≥12 | T. Dapsone VS. T. Vehicle | 5% twice daily, for 12 months | Dapsone gel was a safe and effective for long-term treatment of AV with a rapid onset of its action |
| Anjum, et al. ^(3)^  2023 | Acne Vulgaris  CT  350 | Mean = 23 | T. Dapsone | 5% twice daily, for 12 weeks | Dapsone gel 5% twice daily was an effective, safe, and well-tolerated treatment for AV. |
| Iftikhar, et al. ^(4)^ 2025 | Acne Vulgaris  RCT  110 | 12 - 30  Dapsone mean = 21.75 (4)  Clindamycin mean= 21. 49 (4.34) | T. Dapsone VS T. Clindamycin | 5% once daily, for 12 weeks | Dapsone 5% gel outperformed clindamycin phosphate 1% gel in efficacy, with the additional benefit of once-daily dosing for improved patient convenience. |
| Stein Gold, et al. ^(5)^  2016 | Acne Vulgaris  RCT  2102 | ≥12, Mean = 20.0 (7.47) | T. Dapsone VS. T. Vehicle | 7.5% once daily, for 12 weeks | Dapsone gel 7.5% once daily was an effective, safe, and well-tolerated treatment for AV. |
| Eichenfield, et al. ^(6)^  2016 | Acne Vulgaris  RCT  2238 | ≥12, Mean = 20.4 (7.77) | T. Dapsone VS. T. Vehicle | 7.5% once daily, for 12 weeks | Dapsone gel 7.5% once daily was an effective, safe, and well-tolerated treatment for AV. |
| Thiboutot, et al. ^(7)^  2016 | Acne Vulgaris  RCT  4340 | Dapsone: 12-63  Mean = 20.3 (7.8)  Vehicle: 12-54  Mean= 20.2 (7.5) | T. Dapsone VS. T. Vehicle | 7.5% once daily, for 12 weeks | The safety and tolerability of Dapsone gel 7.5% once daily were reported similar to Dapsone gel 5% twice daily. |
| Tanghetti, E. A., et al. ^(8)^  2018 | Acne Vulgaris  RCT  2160 | ≥12 | T. Dapsone VS. T. Vehicle | 7.5% once daily, for 12 weeks | Dapsone gel 7.5% was efficacious for AV, regardless of baseline lesions count & tends to be more effective in females. |
| Al-Mosawi, et al. ^(9)^  2022 | Acne Vulgaris  RCT  60 | Young | T. Dapsone VS. T. Clindamycin VS. T. Erythromycin VS. Placebo | 5% twice daily, for 12 weeks | Dapsone gel & clindamycin were better than erythromycin in reducing all kinds of lesions and having a lower rate of adverse events. |
| Stockton, et al. ^(10)^  2018 | Acne Vulgaris  Clinical Experience  8 | Mean = 20.6 | T. Dapsone | 7.5% once daily, for 12 weeks | Dapsone gel was effective and well tolerated, according to photographs, dermatologist reports, and patient commentaries. |
| Del Rosso, et al. ^(11)^  2018 | Truncal Acne Vulgaris  CT  20 | ≥12 | T. Dapsone | 7.5% once daily, for 16 weeks | Dapsone gel 7.5% had an acceptable efficacy and safety for truncal AV. |
| Del Rosso, et al. ^(12)^  2015 | Acne Vulgaris  RCT  781 | Adolescent = 12-17, Mean = 14 & Adult = ≥18, Mean = 27 | T. Dapsone VS. T. Vehicle | 5% twice daily, for 12 weeks | Dapsone gel was an effective, safe, and well-tolerated treatment for AV & tended to be more effective in adults. |
| Draelos, et al. ^(13)^  2017 | Acne Vulgaris  RCT  4340 | ≥12, Mean = 20.3 | T. Dapsone VS. T. Vehicle | 7.5% once daily, for 12 weeks | Dapsone gel tended to be more effective in older age and female sex. |
| Lynde and Andriessen ^(14)^  2014 | Acne Vulgaris  Cohort  101 | ≥18, Mean = 31.1 | T. Dapsone | 5% twice daily, for 12 weeks | Treatment success was 69.4% in females' facial acne and minimally irritating. |
| Moore, et al. ^(15)^  2021 | Acne Vulgaris  CT  100 | 9-11, Mean = 10.4 | T. Dapsone | 7.5% once daily, for 12 weeks | Dapsone gel 7.5%, once daily, was a safe, effective, and well-tolerated option in preadolescent AV patients. |
| Raimer, et al. ^(16)^  2008 | Acne Vulgaris  RCT  1306 | 12-15 | T. Dapsone VS. T. Vehicle | 5% twice daily, for 12 weeks | Dapsone in 12 weeks and 12 months had clinically significant efficacy in reducing lesion counts and acceptable safety in adolescents. |
| Alexis, et al. ^(17)^  2016 | Acne Vulgaris  RCT  68 | ≥18 | T. Dapsone | 5% twice daily, for 12 weeks | Dapsone gel was an effective, safe, and well-tolerated treatment for AV in females with a skin of color. |
| Taylor, et al. ^(18)^  2018 | Acne Vulgaris  RCT  4327 | ≥12 | T. Dapsone VS. T. Vehicle | 7.5% once daily, for 12 weeks | Once-daily use of 7.5% Dapsone gel was safe, effective, and well tolerated among various groups of skin phototypes. |
| El-Kashlan, et al. ^(19)^  2024 | Acne Vulgaris  CT  20 | ≥18 | T. Dapsone | 7.5% once daily, for 24 weeks | Once-daily use of 7.5% Dapsone gel was safe, effective, and well tolerated among various groups of skin phototypes. |
| Belum, et al. ^(20)^  2017 | Cetuximab-induced acneiform rash  RCT  11 | NM | T. Dapsone + S. Minocycline | 5% twice daily, for 4 weeks | Their result was statistically insignificant. |
| Piette, et al. ^(21)^  2008 | Acne Vulgaris  RCT  64 | 12-61, Mean = 28 (10) | Two phases of treatment for each individual: T. Dapsone + T. Vehicle | 5% twice daily, for 28 weeks | Dapsone gel was a safe treatment for AV. During treatment, hemolytic anemia proposed to be remote for all patients, including those with G6PD deficiency. |
| Faghihi, et al. ^(22)^  2014 | Acne Vulgaris  RCT  58 | 18-25, Mean = 20.3 | T. Dapsone + S. Isotretinoin VS. S. Isotretinoin | 5% twice daily, for 12 weeks | Dapsone + isotretinoin compared to alone isotretinoin, could reduce the lesion count more, but did not alter the final GAAS score. |
| Hasanbeyzade, et al. ^(23)^  2025 | Acne Vulgaris  Retrospective study  82 | 12–40  Dapsone mean = 21.59 (6.63)  Clindamycin mean = 22.34 (7.66) | T. Dapsone + T Tretinoin VS. T. Clindamycin + T. Tretinoin | 5% once daily, at least 12 weeks | Dapsone and tretinoin compared to clindamycin and tretinoin was more effective. |
| Kircik, L. H. ^(24)^  2016 | Acne Vulgaris  CT  32 | Mean = 24.8 (10.9) | T. Dapsone + S. Doxycycline | 5% twice daily, for 24 weeks | Doxycycline hyclate 100 mg/daily + Dapsone was an effective and well-tolerated regimen to treat moderate to severe AV. Also, Dapsone gel was effective for maintaining a therapeutic response. |
| Darjani, et al. ^(25)^  2022 | Acne Vulgaris  RCT  60 | Mean = 21.85 (2.45) | T. Dapsone + S. Doxycycline VS. T. Benzoyl peroxide + S. Doxycycline | 5% once daily, for 12 weeks | Both groups had a similar efficacy, with a higher significant reported of adverse events in Dapsone group. |
| Hasanbeyzade, et al. ^(26)^  2024 | Acne Vulgaris  Retrospective study  101 | Dapsone mean = 22.38 (7.94)  Benzoyl peroxide mean = 20.53 (8.18) | T. Dapsone + VS. T. Benzoyl peroxide 10% | 5% once daily, NM | Dapsone is effective in the treatment of mild to moderate acne and is  safe in terms of side effects compared to topical 10% benzoyl peroxide |
| Tanghetti, et al. ^(27)^  2011 | Acne Vulgaris  RCT  171 | Mean = 19.8 (6.7) | T. Dapsone + T. Tazarotene VS. T. Tazarotene | 5% twice daily, for 12 weeks | The combination therapy group had a better response in noninflammatory and total lesion count reduction. |
| Fleischer Jr, et al. ^(28)^  2010 | Acne Vulgaris  RCT  203 | ≥12 | T. Dapsone VS. T. Adapalene VS. T. Dapsone + T. Benzoyl peroxide VS. T. Dapsone + Moisturizer | 5% twice daily, for 12 weeks | Inflammatory lesion count reduction and adverse events within groups were similar. Non-inflammatory lesion count reduction and total count lesion reduction were significantly better in the adapalene group. |
| Grove, et al. ^(29)^  2013 | Acne Vulgaris  RCT  NM | 18-45 | T. Benzoyl peroxide + T. Clindamycin phosphate VS. T. Benzoyl peroxide + T. Clindamycin phosphate VS. T. Dapsone + T. Benzyl peroxide + Adapalene | 5% twice daily, for 2 weeks | The Dapsone regime showed more adverse events compared to other parallel groups. |
| Gharib, et al.  ^(30)^  2024 | Acne Vulgaris  RCT  28 | Mean = 20 (2.18) | T. Dapsone VS. T. Spironolactone | 5% twice daily, for 12 weeks | Both regimens had therapeutic effects but were statistically significant in favor of the T. Spironolactone. |
| RCT: Randomized Clinical Trial  CT: Clinical Trial  VS: versus  T. at the initiation of the drug name: Topical formulation of the drug  S. at the initiation of the drug name: Systemic formulation of the drug  NM: Not mentioned | | | | | |

1. Draelos ZD, Carter E, Maloney JM, Elewski B, Poulin Y, Lynde C, Garrett S. Two randomized studies demonstrate the efficacy and safety of dapsone gel, 5% for the treatment of acne vulgaris. J Am Acad Dermatol. 2007;56(3):439.e1-10.

2. Lucky AW, Maloney JM, Roberts J, Taylor S, Jones T, Ling M, Garrett S. Dapsone gel 5% for the treatment of acne vulgaris: safety and efficacy of long-term (1 year) treatment. J Drugs Dermatol. 2007;6(10):981-7.

3. Anjum R, Majeed R, Dawood N, Shahzadi S, Sheikh FZ, Sheikh M. Efficacy and side effects estimation of 5% dapsone gel in the treatment of mild to moderate acne vulgaris. J Pak Assoc Dermatol. 2023;33(4):1524-8.

4. Iftikhar A, Luqman N, Mubeen S, Tariq M, Khalid H, Naseer K. Comparison of the Efficacy of 5% Dapsone Gel and 1% Clindamycin Phosphate Gel in the Treatment of Mild to Moderate Acne Vulgaris. J Pak Assoc Dermatol. 2025;35(1):20-6.

5. Stein Gold LF, Jarratt MT, Bucko AD, Grekin SK, Berlin JM, Bukhalo M, et al. Efficacy and Safety of Once-Daily Dapsone Gel, 7.5% for Treatment of Adolescents and Adults With Acne Vulgaris: First of Two Identically Designed, Large, Multicenter, Randomized, Vehicle-controlled Trials. Journal of Drugs in Dermatology. 2016;15(5):553-61.

6. Eichenfield LF, Lain T, Frankel EH, Jones TM, Chang-Lin JE, Berk DR, et al. Efficacy and Safety of Once-Daily Dapsone Gel, 7.5% for Treatment of Adolescents and Adults With Acne Vulgaris: Second of Two Identically Designed, Large, Multicenter, Randomized, Vehicle-Controlled Trials. Journal of Drugs in Dermatology. 2016;15(8):962-9.

7. Thiboutot DM, Kircik L, McMichael A, Cook-Bolden FE, Tyring SK, Berk DR, et al. Efficacy, safety, and dermal tolerability of dapsone gel, 7.5% in patients with moderate acne vulgaris: A pooled analysis of two phase 3 trials. Journal of Clinical and Aesthetic Dermatology. 2016;9(10):18-27.

8. Tanghetti E, Harper J, Baldwin H, Kircik L, Bai Z, Alvandi N. Once-Daily Topical Dapsone Gel, 7.5%: Effective for Acne Vulgaris Regardless of Baseline Lesion Count, With Superior Efficacy in Females. J Drugs Dermatol. 2018;17(11):1192-8.

9. Al-Mosawi ROA, Hassan JK, Al-Tameemi FF. Comparative Evaluation of Topical Antibiotics in Treatment of Mild to Moderate Acne Vulgaris. International Journal of Drug Delivery Technology. 2022;12(4):1924-7.

10. Stockton TC, Tanghetti EA, Lain E, Zeichner JA, Alvandi N. Clinical Experience With Once-Daily Dapsone Gel, 7.5% Monotherapy in Patients With Acne Vulgaris. Journal of drugs in dermatology : JDD. 2018;17(6):602-8.

11. Del Rosso JQ, Kircik L, Tanghetti E. Management of truncal acne vulgaris with topical dapsone 7.5% gel. Journal of Clinical and Aesthetic Dermatology. 2018;11(8):45-50.

12. Del Rosso JQ, Kircik L, Gallagher CJ. Comparative efficacy and tolerability of dapsone 5% gel in adult versus adolescent females with acne vulgaris. The Journal of clinical and aesthetic dermatology. 2015;8(1):31-7.

13. Draelos ZD, Rodriguez DA, Kempers SE, Bruce S, Peredo MI, Downie J, et al. Treatment Response With Once-Daily Topical Dapsone Gel, 7.5% for Acne Vulgaris: Subgroup Analysis of Pooled Data from Two Randomized, Double-Blind Studies. Journal of Drugs in Dermatology. 2017;16(6):591-8.

14. Lynde CW, Andriessen A. Cohort study on the treatment with dapsone 5% gel of mild to moderate inflammatory acne of the face in women. SKINmed. 2014;12(1):15-21.

15. Moore AY, Lain EL, McMichael A, Kircik L, Zaenglein AL, Hebert AA, Grada A. Once-daily Dapsone 7.5% Gel for the Treatment of Acne Vulgaris in Preadolescent Patients: A Phase IV, Open-label, 12-week Study. J Clin Aesthet Dermatol. 2021;14(4):43-8.

16. Raimer S, Maloney JM, Bourcier M, Wilson D, Papp K, Siegfried E, Garrett S. Efficacy and safety of dapsone gel 5% for the treatment of acne vulgaris in adolescents. Cutis. 2008;81(2):171-8.

17. Alexis AF, Burgess C, Callender VD, Herzog JL, Roberts WE, Schweiger ES, et al. The Efficacy and Safety of Topical Dapsone Gel, 5% for the Treatment of Acne Vulgaris in Adult Females With Skin of Color. J Drugs Dermatol. 2016;15(2):197-204.

18. Taylor SC, Cook-Bolden FE, McMichael A, Downie JB, Rodriguez DA, Alexis AF, et al. Efficacy, Safety, and Tolerability of Topical Dapsone Gel, 7.5% for Treatment of Acne Vulgaris by Fitzpatrick Skin Phototype. Journal of Drugs in Dermatology. 2018;17(2):160-7.

19. El-Kashlan N, Cices A, Kaufman B, Rosa JCD, Sanabria-Gonzalez I, Khattri S, Alexis A. An Open-label Study to Investigate the Efficacy and Tolerability of Dapsone Gel, 7.5% in the Treatment of Acne Vulgaris in Men and Women With Skin of Color. Journal of Drugs in Dermatology. 2024;23(6):410-7.

20. Belum VR, Marchetti MA, Dusza SW, Cercek A, Kemeny NE, Lacouture ME. A prospective, randomized, double-blinded, split-face/chest study of prophylactic topical dapsone 5% gel versus moisturizer for the prevention of cetuximab-induced acneiform rash. J Am Acad Dermatol. 2017;77(3):577-9.

21. Piette WW, Taylor S, Pariser D, Jarratt M, Sheth P, Wilson D. Hematologic safety of dapsone gel, 5%, for topical treatment of acne vulgaris. Arch Dermatol. 2008;144(12):1564-70.

22. Faghihi G, Rakhshanpour M, Abtahi-Naeini B, Nilforoushzadeh MA. The efficacy of 5% dapsone gel plus oral isotretinoin versus oral isotretinoin alone in acne vulgaris: A randomized double-blind study. Advanced biomedical research. 2014;3:177-.

23. Hasanbeyzade S, Şenel E. Comparison of topical dapsone + tretinoin and clindamycin + tretinoin combination in terms of effectiveness in the treatment of mild and moderate acne vulgaris: a retrospective analysis. Archives of Dermatological Research. 2025;317(1).

24. Kircik LH. Use of Dapsone 5% Gel as Maintenance Treatment of Acne Vulgaris Following Completion of Oral Doxycycline and Dapsone 5% Gel Combination Treatment. Journal of Drugs in Dermatology. 2016;15(2):191-5.

25. Darjani A, Aboutaleb E, Alizadeh N, Rafiei R, Nejad KG, Nabatchii S, et al. Efficacy, safety, and tolerability of dapsone 5% gel and benzoyl peroxide 5% gel in combination with oral doxycycline in treating moderate acne vulgaris: A randomized clinical trial. Iranian Journal of Dermatology. 2022;25(2):132-41.

26. Hasanbeyzade S, Şenel E. Efficacy and Tolerability of Topical Dapsone vs Benzoyl Peroxide in Mild to Moderate Acne Vulgaris Treatment: A Retrospective Study. Duzce Medical Journal. 2024;26(3):198-202.

27. Tanghetti E, Dhawan S, Green L, Ling M, Downie J, Germain MA, et al. Clinical Evidence for the Role of a Topical Anti-Inflammatory Agent in Comedonal Acne: Findings From a Randomized Study of Dapsone Gel 5% in Combination With Tazarotene Cream 0.1% in Patients With Acne Vulgaris. Journal of Drugs in Dermatology. 2011;10(7):783-92.

28. Fleischer Jr AB, Shalita A, Eichenfield LF, Abramovits W, Lucky A, Garrett S, et al. Dapsone gel 5% in combination with adapalene gel 0.1%, benzoyl peroxide gel 4% or moisturizer for the treatment of acne vulgaris: a 12-week, randomized, double-blind study. J Drugs Dermatol. 2010;9(1):33-40.

29. Grove G, Zerweck C, Gwazdauskas J. Tolerability and irritation potential of four topical acne regimens in healthy subjects. J Drugs Dermatol. 2013;12(6):644-9.

30. Gharib K, Samir M, Mohamed G, Rageh MA. Efficacy and safety of topical spironolactone versus topical dapsone in the treatment of acne vulgaris. Archives of Dermatological Research. 2024;316(10).
